# Combined Hybridization and Evaluation of High-Lysine Rice: Nutritional and Physicochemical Qualities and Field Performance

**DOI:** 10.3390/ijms232012166

**Published:** 2022-10-12

**Authors:** Ying Ye, Yan Tan, Yuquan Meng, Qi You, Dongsheng Zhao, Qingqing Yang

**Affiliations:** 1 Key Laboratory of Plant Functional Genomics of the Ministry of Education/Jiangsu Key Laboratory of Crop Genomics and Molecular Breeding/Jiangsu Co-Innovation Center for Modern Production Technology of Grain Crops/Jiangsu Key Laboratory of Crop Genetics and Physiology, College of Agriculture, Yangzhou University, Yangzhou 225009, China; 2 Department of Biotechnology, School of Life Science and Technology, University of Electronic Science and Technology of China, Chengdu 610054, China

**Keywords:** rice, lysine, amino acid, physicochemical quality, field performance

## Abstract

Rice, as a major food crop, provides necessary energy and nutrition for humans and livestock. However, its nutritional value is affected by lysine. Using point mutation, we previously obtained *AK2* (aspartokinase) and *DHDPS1* (dihydrodipicolinate synthase) genes insensitive to lysine feedback inhibition and constructed transgenic lines AK2-52 and DHDPS1-22, which show increased lysine synthesis, as well as Ri-12, which shows decreased lysine degradation by inhibiting rice lysine ketoglutarate reductase/saccharopine dehydrogenase (LKR/SDH) activity. In this study, further transgenic lines were hybridized and evaluated. The lysine content of mature seeds from pyramid lines PRD and PRA increased 32.5- and 29.8-fold, respectively, compared with the wild-type, while the three-gene pyramiding line PRDA had a moderate lysine content. The total lysine, total free lysine, and total protein contents of PRD and PRA also increased and had no obvious impact on the physical and chemical quality, seed appearance, and main agronomic traits. Meanwhile, comparative analysis with polygenic polymeric lines GR containing bacterial *AK* (*lysC*) and *DHDPS* (*dapA*) genes revealed differences in the way bacterial and endogenous rice AK and DHDPS regulate lysine biosynthesis. These results provide a reference for further evaluation and commercialization of high-lysine transgenic rice.

## 1. Introduction

Ensuring food security is the key to overcoming world hunger and malnutrition. Innovations in plant breeding and effective use of crop biotechnology play a crucial role in simultaneously solving the problems of food and nutrition security linked to the COVID-19 pandemic and the risk of growing global hunger associated with the rapid growth target of 10 billion people by 2050 [1]. Rice, as an important food crop, contains high-quality protein that is easily digestible, providing essential energy and nutrients for both humans and livestock [2]. However, its nutritional value is limited by its relatively low content of lysine, the first limiting amino acid in rice, which prevents efficient absorption and utilization of rice protein. In turn, this has a negative impact on the overall dietary structure, resulting in malnutrition and other diseases [3]. Strategies aimed at increasing the content of lysine in crops in order to improve their nutritional quality are thus required.

Lysine enhancement in cereal crops has been studied for more than 50 years, although few quantitative trait loci (QTLs) and high-lysine mutants have yet been detected. Despite the successful breeding of o2 mutants and quality protein maize (QPM), limited progress has been made in terms of increasing the lysine content in other QTLs and mutants [4]. However, with the development of new molecular biotechnologies and the clarification of aspartate metabolism in plants, direct regulation of lysine metabolism through metabolic engineering has become a feasible strategy.

The lysine metabolic pathway in higher plants branches from the aspartate pathway, separate branches of which also regulate the biosynthesis of threonine, isoleucine, and methionine [5]. A number of studies have shown that lysine biosynthesis and degradation are controlled by various branching enzymes, of which aspartokinase (AK) and dihydrodipicolinic acid synthase (DHDPS) have the greatest impact [5]. AK is the first key enzyme in the aspartate pathway and is subject to concerted feedback inhibition of lysine and threonine [5]. The molecular properties of AK have been determined in both microorganisms and plants [5]. For example, in *Escherichia coli*, AKIII is encoded by the *lysC* gene, the specificity of which is subject to lysine feedback inhibition. Meanwhile, Shaul et al. (1992) isolated a *lysC* allele insensitive to lysine feedback inhibition and transformed it into tobacco, improving the activity of AK and the accumulation of free threonine [6]. AK has at least two isoenzyme forms in plants, one of which is subject to lysine feedback inhibition and the other to threonine feedback inhibition [5]. Compared with AK, DHDPS is subject to specific lysine feedback inhibition in plants and is the first major rate-limiting enzyme in the branched pathway of lysine biosynthesis. However, early studies showed that DHDPS in bacteria is significantly less sensitive to lysine than DHDPS in plants [7]. Lysine-insensitive or -hyposensitive mutant bacterial AK and DHDPS have thus been applied to plants to improve free lysine levels [7]. Natural and induced mutants of lysine-insensitive AK and/or DHDPS (showing resistance to the lysine analog S-(2-aminoethyl)-cysteine) have also been found in plants, revealing single amino acid changes in their sequences [8]. However, these mutants, especially in crops, show slightly increased levels of lysine [4]. For example, Yang et al. (2021) recently modified the cDNA encoding AK and DHDPS in rice using overlapping polymerase chain reaction (PCR), obtaining two AK mutants and five DHDPS mutants with reduced sensitivity to lysine feedback inhibition [9]. Upon introduction into rice, they significantly increased the lysine content of the seeds.

Lysine-ketoglutarate reductase/saccharopine dehydrogenase (LKR/SDH) also plays a key role in the catabolism and accumulation of lysine in plants [5]. Regulation of lysine catabolism was previously achieved by inhibiting or knocking out LKR activity, while co-expression with AK or DHDPS expressing reduced sensitivity to lysine also allowed regulation of the biosynthetic pathway, resulting in a series of engineered crops, all with significantly increased levels of lysine in mature seeds [10,11]. In maize, overexpression of the bacterial *DHDPS* gene showing insensitivity to lysine feedback inhibition with the LKR-RNAi structure significantly improved free lysine levels in the seeds [10,12]. Meanwhile, in rice, simultaneous overexpression of bacterial *AK* and *DHDPS* genes, as well as inhibition of *LKR/SDH* gene expression via a CaMV 35S promoter, increased free lysine levels in the mature transgenic rice seeds 25-fold compared with the wild-type (WT) [11]. Moreover, free lysine levels in mature transgenic rice seeds increased 58.5-fold compared with the WT following combined expression of endogenous mutant *AK2* and *DHDPS1* with *LKR*-RNAi [9].

However, studies suggest that increased levels of lysine also have an effect on plant metabolism, as well as subsequent growth and development [7]. For example, in rice, overexpression of *DHDPS* showing reduced sensitivity to maize lysine feedback inhibition resulted in a fourfold increase in free lysine in the mature seeds as well as subsequent morphological changes [13]. Moreover, overexpression of bacterial *AK* and *DHDPS* in rape and soybean resulted in shrunken seeds, low germination rates, and other abnormal phenotypes [7]. Overexpression of bacterial lysine-insensitive *DHDPS* and inhibition of *LKR* gene expression also led to delayed germination of Arabidopsis seeds [14]. Furthermore, overexpression of bacterial *AK* and *DHDPS* and inhibition of rice *LKR* gene expression resulted in mature high-lysine rice seeds with a brown-black appearance [11]. In contrast, there were no obvious changes in the mature seeds following overexpression of endogenously mutated *AK2* and *DHPDS1* [9]. Subsequent studies suggested that the above damages of the lysine excessive accumulation to plant growth and development, physiological and biochemical characteristics, and agronomic traits were connected with the tricarboxylic acid cycle, tryptophan metabolism, starch metabolism, unfolded protein response, and abiotic and biotic stress responses [15]. Moreover, expression of the LKR/SDH gene is induced by the plant stress hormone abscisic acid and methyl jasmonate [16]. Synergistically with the defense hormone salicylic acid, the lysine metabolic pathway has a key role in plant systemic acquired resistance (SAR) to pathogen infection in Arabidopsis [17]. In contrast, Yang et al. (2018) found the lysine metabolism inducing the jasmonate signaling pathway and TDC expression in rice, coinciding with serotonin accumulation and dark-brown pigmentation [18]. Xu et al. (2022) found that high ethylene level impedes amino acid biosynthesis in rice grains and reduces the content of some amino acids including lysine [19]. Thus, not only does lysine metabolism involve a complex regulatory network, but it may also vary among species [7,15]. Recently, the role of micronutrient-lysine chelates in reducing Cr or Cd toxicity and improving plant growth in crops by regulating antioxidant enzyme activities has been revealed [20,21,22]. This may relieve the pressure of lysine redundancy on plants to some extent.

Tissue-specific promoters can be used to specifically improve crop quality in plant genetic engineering, while avoiding the adverse effects caused by constitutive expression. The characteristics of the rice endosperm-specific promoter *Gt1* are well known, thus it is widely used in rice genetic engineering, especially in the improvement of edible endosperm traits [23]. We previously expressed respective mutant *AK2* and *DHDPS1* in rice using the *Gt1* promoter, significantly increasing the free lysine content of the ripe endosperm [9]. In this study, we further hybridized the Gt1-driven *AK2*, *DHDPS1*, and *LKR*-RNAi structures and screened high-lysine rice in order to evaluate the resulting field agronomic traits, as well as physical and chemical qualities.

## 2. Results

### 2.1. Hybrid Breeding of High-Lysine Rice

**Figure 1:**
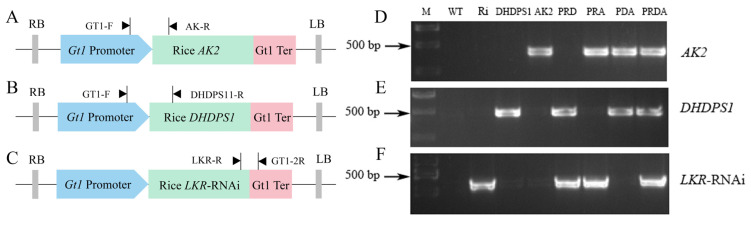
PCR analysis of target genes in the transgenic rice plants. (**A**–**C**) Construction of the recombinant vector and detection primers. Detection of (**D**) the *AK* gene, (**E**) the *DHDPS* gene, and (**F**) *LKR*-RNAi structure in the transgenic and wild-type (WT) rice.

We previously employed point mutation to obtain *AK2* and *DHDPS1* mutants showing reduced sensitivity or insensitivity to lysine feedback inhibition, with expression in the endosperm driven by the rice endosperm-specific promoter *Gt1* [9]. In this study, to further increase the lysine content of the endosperm, we combined the *LKR*-RNAi structure with *AK2* and *DHDPS1* (all driven by the *Gt1* promoter) to obtain multigene pyramiding PRD, PRA, and PRDA (Figure 1A–C; Supplementary Table S1). Stable transgenic plants were subsequently obtained after more than three consecutive generations of PCR screening (Figure 1D–F).

### 2.2. Molecular Identification of High-Lysine Pyramiding Lines

**Figure 2:**
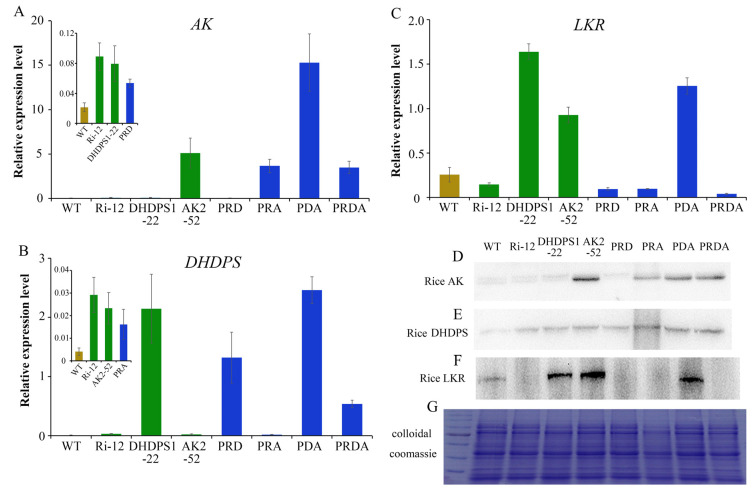
Expression analysis of AK, DHDPS, and LKR genes in developed rice seeds from the transgenic and wild-type (WT) rice. (**A**–**C**) Transcription level analysis of AK, DHDPS, and LKR genes in developed seeds obtained 15 days after flowering (DAF). (**D**–**F**) Western blot analysis of AK, DHDPS, and LKR proteins in developed seeds obtained 15 DAF. (**G**) SDS-PAGE analysis of total protein contents.

Total RNA was isolated from developing seeds of transgenic rice and WT plants 15 days after flowering (DAF) and used for the analysis of transcriptional levels of *AK*, *DHDPS*, and *LKR* target genes. Expression levels of *AK* and *DHDPS* in seeds of AK2-52, DHPDS1-22, and their pyramid line PDA increased significantly, while expression of the key gene *LKR* was also significantly increased compared with the WT (Figure 2A–C). This was possibly due to an increase in the lysine biosynthetic pathway, which induced improvements in the degradation pathway. Expression levels of *AK* and *DHDPS* in transgenic rice containing all three genes were also significantly higher than in the WT, but they were lower than those in the AK2-52 and DHPS1-22 transgenic lines (Figure 2A,B). *LKR* gene expression was significantly lower in all transgenic lines and pyramid lines containing the *LKR*-RNAi structure, suggesting that the LKR gene was effectively inhibited (Figure 2C).

To further verify the changes in protein levels of the target genes, total protein was extracted from seeds collected at 15 DAF and subjected to Western blotting. Overall, protein expression in transgenic rice with a single gene and their pyramid lines was essentially in agreement with the transcriptional levels (Figure 2D–G), suggesting that AK2 and DHDPS1 were effectively transferred and expressed, while LKR was correspondingly inhibited.

### 2.3. Rice AK2/DHDPS1 Significantly Increased the Lysine Content and Was Superior to Bacterial lysc/dapA in the Rice Endosperm

**Figure 3:**
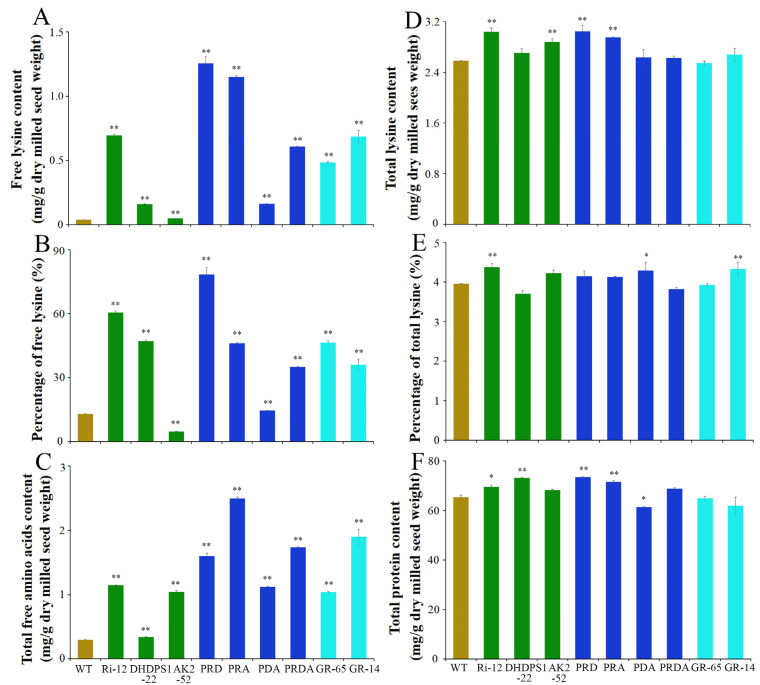
Analysis of lysine, total free amino acids, and total protein in mature milled rice flour from the transgenic and WT rice. (**A**) The free lysine content (mg/g dry milled seed weight) and (**B**) percentage of free lysine to total free amino acids (g/100 g dry milled seed weight). (**C**) The total free lysine content (mg/g dry milled seed weight) and (**D**) total lysine content (mg/g dry milled seed weight). (**E**) The percentage of total lysine to total protein (g/100 g dry milled seed weight). (**F**) The crude protein content (mg/g dry milled seed weight). “*” indicates significant difference (*p* < 0.05), “**” indicates extremely significant difference (*p* < 0.01).

Amino acid and protein levels were subsequently compared in mature milled seeds of transgenic and WT plants. The free lysine content of AK2-52, DHDPS1-22, and their pyramid line PDA was limited, while that of Ri-12 and its hybrid lines increased significantly compared with the WT. Notably, the free lysine contents of the two-gene pyramid lines PRD and PRA increased 32.5- and 29.8-fold, respectively, compared with the WT, and were higher than those of the Gt1-driven transgenic lines GR-14 and GR-65, which combined bacterial *AK* and *DHDPS* with rice *LKR*-RNAi (Figure 3A). However, the free lysine content of the multigene pyramid line PRDA, which simultaneously contained Ri-12, AK2-52, and DHDPS1-22, was higher than that of the WT, but not that of the parents (Figure 3A). These findings suggest that regulation of the lysine biosynthetic pathway plays a limited role in increasing the free lysine content of rice, although its effect is significantly improved when combined with the lysine degradation pathway. This is consistent with the previous conclusion [24]. In addition, total free amino acid levels also increased significantly with the increase in free lysine levels, especially in the pyramid lines PRD and PRA, with increases of 5.4- and 8.4-fold, respectively, compared with the WT (Figure 3C). Moreover, the percentage of free lysine to total free amino acids in mature milled seeds of the PRD transgenic line reached 78.3% (Figure 3B).

In addition, the total lysine content was significantly enhanced in the high free-lysine transgenic rice, with increases of 18.1 and 14.4% in the PRD and PRA mature milled seed, respectively, compared with the WT (Figure 3D). The protein content also increased in all transgenic lines (Figure 3F), with 12.5 and 9.5% increases in PRD and PRA, respectively, compared with the WT. Corresponding increases in the proportion of total lysine to protein were also observed in the high free-lysine transgenic lines, thereby optimizing the proportion of lysine (Figure 3E,F). Taken together, these findings suggest that combined expression of rice *AK2*/*DHDPS1* genes showing reduced sensitivity to lysine feedback inhibition with the *LKR*-RNAi structure greatly enhanced the lysine content and nutritional quality of the rice endosperm. Moreover, the effect was greater than that of the similar bacterial *AK* and *DHDPS* genes in rice.

### 2.4. Effects of Lysine Enrichment on Other Amino Acids

**Figure 4:**
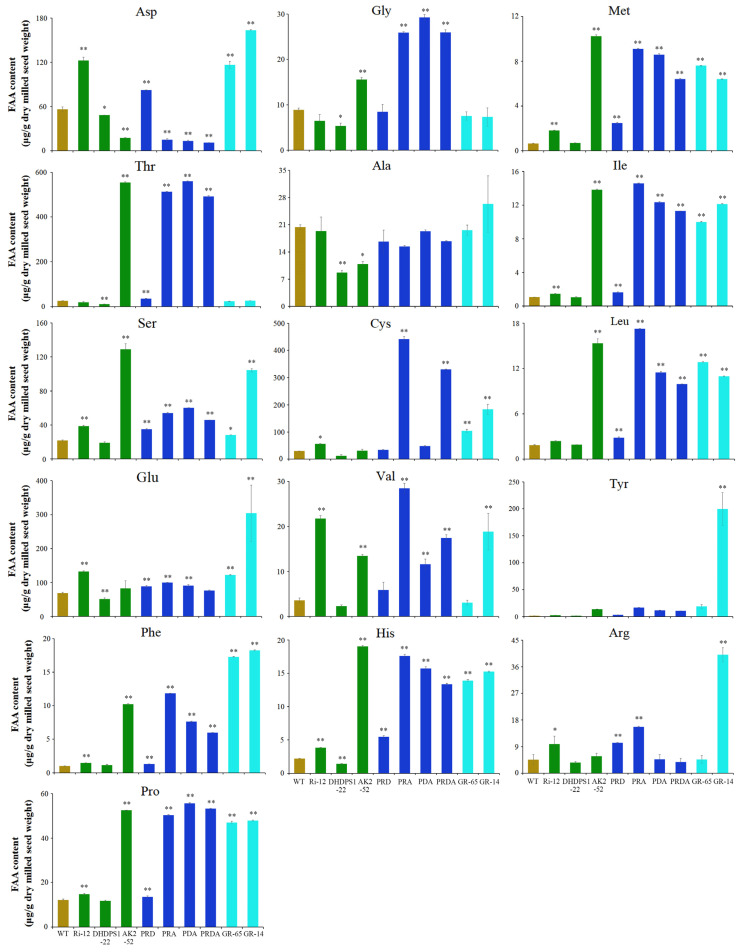
Contents of other free amino acids analysis in mature milled rice flour from the transgenic and WT rice. “*” indicates significant difference (*p* < 0.05), “**” indicates extremely significant difference (*p* < 0.01).

Higher plants possess a complex regulatory network of free amino acids, with various amino acids cooperating to maintain metabolic balance. In this study, mature milled seeds of the AK2-52 transgenic line and its hybrid lines showed a significant increase in downstream amino acids threonine, serine, glycine, methionine, leucine, isoleucine, and valine, all of which are regulated by AK (Figure 4). Visible changes in other amino acids were also observed. Interestingly, however, in the DHDPS1-22 transgenic line and its hybrid PRD (lacking AK2-52), these additional amino acids were less affected (Figure 4). Overall, these findings suggest that regulating expression of the *AK* gene has a greater impact on the aspartate metabolic pathway than regulating expression of the *DHDPS* gene.

Meanwhile, the pyramid line PRDA also showed differential changes in amino acid levels compared with the transgenic lines GR-14 and GR-65, which contained bacterial *AK* and *DHDPS*. For example, Thr and Gly were significantly higher in the PRDA transgenic line compared with the WT, with no obvious differences in GR-14 and GR-65 (Figure 4). These findings suggest that rice endogenous *AK* and *DHDPS* act differently to bacterial *AK* and *DHDPS* in regulating amino acid biosynthesis, transport, and accumulation in rice.

### 2.5. Effect of Lysine Enrichment on the Physicochemical Quality of Milled Rice 

As shown in Supplementary Figure S1, there was no obvious impact on the shape of mature transgenic rice seeds following specific expression of *AK2* and *DHDPS1*, with the *LKR*-RNAi structure (Supplementary Figure S1). However, transgenic lines showing high expression of the *AK* gene had greater chalkiness (Supplementary Figure S1).

**Figure 5:**
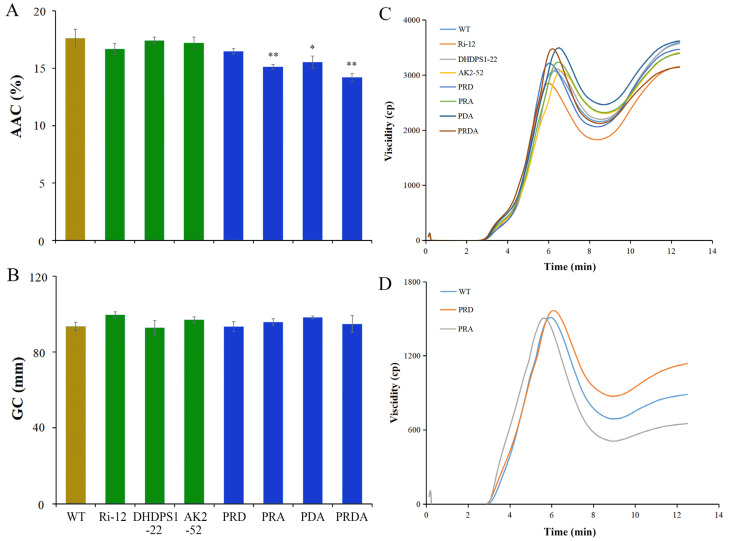
Physical and chemical quality analysis of the transgenic and WT rice. (**A**) The apparent amylose content (g/100 g) and (**B**) gel consistency (mm) of mature milled rice flour. (**C**,**D**) RVA spectral analysis of mature milled rice flour and starch. “*” indicates significant difference (*p* < 0.05), “**” indicates extremely significant difference (*p* < 0.01).

As the main compound of the rice endosperm, the content and properties of starch determine overall quality. We thus examined the physicochemical qualities of the transgenic lines. As a result, the apparent amylose content of single-gene transgenic lines was found to be unaltered, while the gel consistency of Ri-12 and AK2-52 was improved. The apparent amylose content of the pyramid lines was reduced, while the corresponding gel consistency improved, leading to a softer texture compared with the WT (Figure 5A,B). The high-lysine line PRD had similar RVA profiles to the WT, while those of the remaining pyramid lines were different, with lower Setback values and a higher Peak time (Figure 5C, Supplementary Table S1). Further RVA spectrum analysis of starch extracted from the high-lysine lines PRD and PRA showed that the RVA spectrum and eigenvalues of the starch were not significantly different from those of the WT (Figure 5D, Supplementary Table S1). These findings suggest that differences in the physical and chemical qualities of rice flour are caused by an increase in amino acid and protein levels. Overall, the results suggest that lysine enrichment has a certain impact on the physical and chemical qualities of rice flour from milled mature seed, but has no or little impact on starch in the pyramid rice.

### 2.6. Field Agronomic Traits in the Transgenic Rice

**Figure 6:**
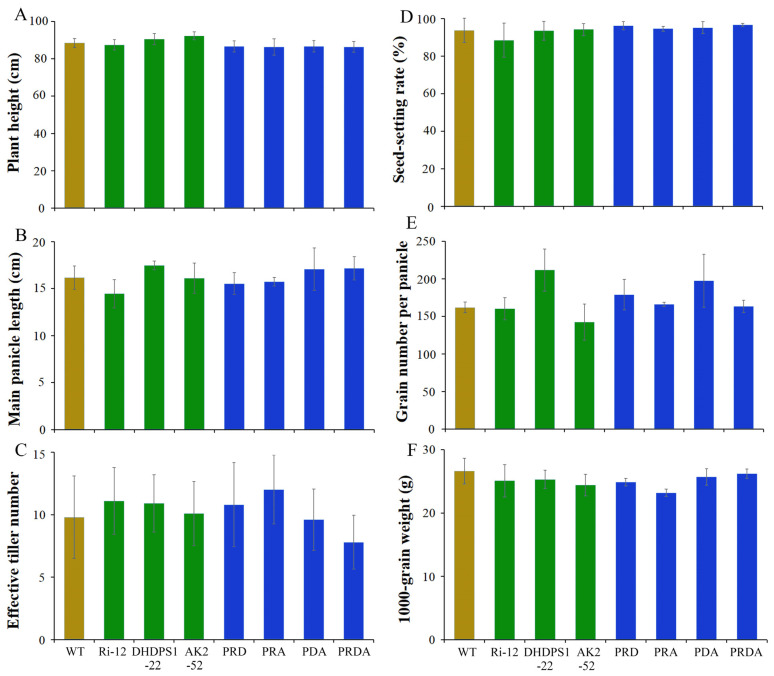
Investigation of the main agronomic traits of the transgenic and WT rice. (**A**) Plant height (cm). (**B**) Main panicle length (cm). (**C**) Effective tiller number. (**D**) Seed-setting rate (%). (**E**) Grain number per panicle. (**F**) 1000-grain weight (g).

All transgenic rice grew well and similar to the WT throughout the growing period. Moreover, comparisons of the main agronomic traits of the transgenic lines and WT after maturity revealed no obvious differences (Figure 6). Grain number per ear in transgenic line DHPDS1-22 was higher than that of the WT (Figure 6E), while the 1000-grain weight of PRA was reduced (Figure 6F), but there was no statistically significant difference. In general, agronomic traits are greatly affected by field planting and the local environment. To verify the effect in the transgenic lines, the main agronomic traits were thus investigated in a subsequent generation. As a result, the agronomic traits of transgenic lines planted in Yangzhou in 2018 were not significantly different from those of the WT (Supplementary Figure S2). Overall, these findings suggest that expression of *AK2*, *DHDPS1*, and *LKR*-RNAi had no substantial impact.

## 3. Discussion

With clarification of the metabolic pathway of aspartate, metabolic engineering technology has become an effective way of improving the lysine content of crops. By regulating the aspartate pathway, lysine content has been successfully increased in soybean, rape, maize, Arabidopsis, and rice [7]. In this study, the free lysine content of transgenic lines AK2-52 and DHDPS1-22 harboring *AK2* and *DHDPS1*, respectively, driven by the rice endosperm-specific promoter Gt1 increased 1.3- and 4.2-fold, while the free lysine content considerably increased in corresponding pyramid lines PRA and PRD generated via hybridization with Ri-12 (Figure 3). These findings suggest that increases in the lysine content require simultaneous regulation of lysine synthesis and catabolism, consistent with previous findings [24]. However, the levels of free lysine in the two-gene transgenic lines PRD and PRA were significantly higher than those in the three-gene pyramid line PRDA (Figure 3A), suggesting that simultaneous regulation of *AK* and *DHDPS* did not affect the lysine biosynthesis pathway. This was possibly caused by the amino acid pool balance, which is required for normal cell growth [25]. Moreover, the lysine content of rice possessing endogenous *AK2*, *DHDPS1*, and *LKR*-RNAi also showed a limited increase compared with transgenic rice containing similar bacterial *AK* and *DHDPS* (Figure 3A,D).

For high-lysine crop breeding, overexpression of *AK2* was found to be more effective at increasing levels of threonine than levels of lysine (Figure 4). Moreover, compared with moderate regulation under *DHDPS* expression, regulation of *AK* expression also had an effect on contents of other amino acids (Figure 4). These findings suggest that metabolic engineering aimed at increased lysine levels should give priority to overexpression of the *DHDPS* gene and inhibition of *LKR*.

Expression of maize-mutated *DHDPS* in rice using the endosperm-specific promoter Gt1 was found to have no effect on the free lysine content of mature seeds [13]. Similar findings were also observed following expression of bacterial DHDPS insensitive to lysine feedback inhibition [26]. In contrast, specific expression of bacterial *DHDPS* in rape and soybean effectively increased the free lysine content of the resulting seeds [27]. Here, specific expression of mutated rice *AK2* or *DHDPS1* in rice also caused a significant increase in the lysine content of the mature endosperm (Figure 3A). These differing findings may have been caused by differences between species or DHDPS characteristics.

Meanwhile, specific expression of bacterial *AK* and *DHDPS* genes insensitive to lysine feedback inhibition in rice had little or no impact on the expression of endogenous rice *AK* and *DHDPS* genes (Supplementary Figure S3), suggesting differences in the expression and regulation of *AK* and *DHDPS* genes between species. In higher plants, lysine, threonine, methionine, and isoleucine all employ aspartate as the shared synthetic precursor, mainly via the aspartate metabolic pathway [5]. AK, as the first rate-limiting enzyme in the aspartate pathway, decomposes aspartate to produce aspartyl phosphate, which respectively enters the lysine and threonine synthesis pathways. In this study, increased expression of AK significantly increased free threonine, lysine, methionine, and isoleucine contents in the mature seeds, as well as contents of the closely related glycine, valine, and leucine, while aspartate decreased accordingly (Figure 4). Unexpectedly, contents of alanine, histidine, arginine, proline, tyrosine, and phenylalanine, all of which are distantly related to the aspartate pathway, varied greatly (Figure 4). These amino acids are all related to plant stress responses, suggesting that the aspartate pathway is involved in multiple metabolic pathways related to plant growth and development, as well as metabolic regulation, playing multiple roles.

Previous studies have shown that excessive accumulation of lysine in plants has adverse effects, resulting in wrinkled seeds, a reduced germination rate, altered oil content, and decreases in yield [7]. Meanwhile, research further suggests that these changes are related to energy metabolism, stress responses, carbon and nitrogen metabolism, and other metabolic pathways [9]. Hence, the regulatory metabolic connections to rate-limiting target genes of lysine metabolism should also be studied to break the barrier of homeostasis, allowing metabolic flow to elevate lysine. The micronutrient-lysine chelates in reducing heavy metal toxicity and improving plant growth in crops may provide a new strategy to reduce the lysine toxicity [20,21,22]. Moreover, the utilization of excellent germplasm resources can also effectively improve the nutritional quality of crops [28,29]. In physiology and ecology, optimized hormone treatment and cultivation techniques are both good choices to increase the lysine content of crops and reduce their toxicity [22,30].

In this study, the main agronomic traits of the transgenic lines and corresponding pyramid lines were not significantly different from those of the WT, which is consistent with previous results in high-lysine rice [9]. However, chalkiness in the transgenic line expressing *AK2* and its corresponding pyramid line increased to varying degrees. As already known, the expression of *AK* gene is regulated by the photosynthesis-related metabolites sucrose and phosphate, and regulates nitrogen and carbon metabolism [31]. In addition, the glycine content also increased in high lysine plants and was found to be the key intermediate metabolite during photorespiration [32]. Thus, this is possibly due to the effect of high AK expression on UPR protein folding or photosynthesis via the aspartate pathway, which in turn affect the formation and levels of chalkiness [31,33]. Meanwhile, the transgenic line overexpressing *DHDPS1* and its corresponding pyramid line PRD did not change significantly, possibly because DHDPS has less impact on other metabolic pathways than AK.

## 4. Materials and Methods

### 4.1. Experimental Materials

The rice materials used in this study were japonica variety Wuxiangjing 9 and seven new transgenic rice lines (Supplementary Table S2). The seven transgenic lines were derived from different DNA structures and combinations as follows: Ri-12 contained the RNAi structure of the rice *LKR* gene driven by the endosperm-specific promoter Gt1; DHPS1-22 contained the Gt1-initiated *DHDPS1* gene (mutated rice *DHDPS* gene); AK2-52 contained the Gt1-initiated *AK2* gene (mutated rice *AK* gene); PRA (Ri-12/AK2-52), PRD (Ri-12/DHDPS1-22), PDA (DHDPS1-22/AK2-52), and PRDA (Ri-12/DHDPS1-22/AK2-52) represent transgenic hybrid polylines obtained from traditional hybrid polymerization of Ri-12, DHDPS1-22, and AK2-52. All transgenic lines were isogenic. They were planted in Yangzhou, Jiangsu, and managed according to conventional planting methods. PCR identification was performed using transgene-specific primers (Supplementary Table S3) according to the method of Yang et al. (2021) [9]. Transgenic lines GR-14 and GR-65 were used for comparative analysis. They were driven by three gene segments from the rice endosperm promoter Gt1 promoter and resulted in overexpression of the two bacterial genes *lysC* (*AK*) and *dapA* (*DHDPS*), as well as a decrease in endogenous *LKR*/*SDH* genes in rice.

### 4.2. Quantitative RT-PCR and Western Blot Analysis

Total RNA was extracted from unripe seeds about 15 DAF using an RNA extraction kit (TAKARA, Tokyo, Japan). The isolated RNA was purified and reverse transcribed into cDNA for quantitative PCR analysis. The specific primers used are listed in Supplementary Table S3. The rice actin gene was used as an internal reference gene and the relative expression of the target gene was analyzed based on the ΔΔCt method [34]. Total protein was extracted from developed seeds obtained 15 DAF, as described previously [24]. Expression levels of the target proteins were detected with Western blotting using rice AK, DHDPS, and LKR-specific antibodies.

### 4.3. Composition and Quality Analysis of Seed Endosperm Compounds

Mature seeds were refined and milled into rice flour for analysis of composition, as well as physical and chemical qualities [35]. A Megazyme kit (Megazyme, Wicklow, Ireland) was used to determine the total starch content [36], while the crude protein content was determined based on the Kjeldahl method. Starch was prepared and purified based on the neutral protease method [36]. The total starch content, protein content, AAC, GC, and RVA contents were analyzed as described previously [35,36]. Total amino acid and free amino acid contents were analyzed according to Yang et al. (2016) [11]. Three biological repeats were designed for each of the above samples.

### 4.4. Investigation of Field Agronomic Traits

Transgenic lines Ri-12, DHDPS1-22, AK2-52, polylines, and corresponding non-transgenic WT were planted in the test field of Yangzhou University from 2017 to 2021, and grown together under the same climatic conditions. Three replicate plots were established, with random planting of test strains in each plot. After reaching maturity, agronomic traits such as plant height, effective tiller number, setting rate, and the 1000-kernel weight were determined.

### 4.5. Statistical Analysis

Sample data described in this experiment were obtained from at least three sets of repeats unless otherwise specified. All presented data represent the mean SD. All data were analyzed based on the Tukey (HSD) test using SPSS software to determine any statistical differences between each transgene and the WT. * represents a statistically significant difference between the transgenic line and the WT (*p* < 0.05) and ** represents a highly statistically significant difference between the transgenic line and the WT (*p* < 0.01).

## Data Availability

The original data for this present study are available from the corresponding authors.

## Supplementary Materials

The following supporting information can be downloaded at https://www.mdpi.com/article/10.3390/ijms232012166/s1.
