# Supplementary material for: Combined Hybridization and Evaluation of High-Lysine Rice: Nutritional and Physicochemical Qualities and Field Performance
Source: Int J Mol Sci. 2022 Oct 12;23(20):12166. doi: 10.3390/ijms232012166 (PMC9603072; doi:10.3390/ijms232012166)
Supplement: Supplementary file 1 [file ijms-23-12166-s001.zip › ijms-1928672-supplementary.pdf]

Supplementary Figures

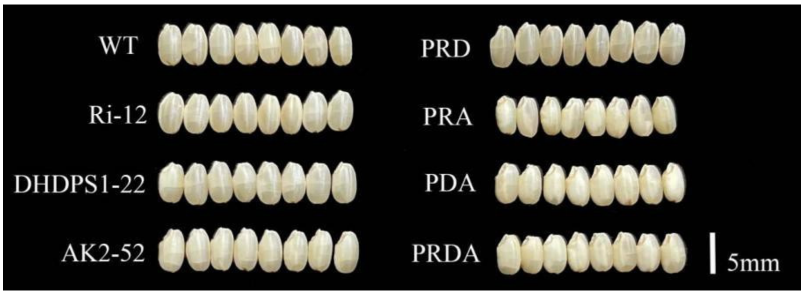

Figure S1. Phenotypes of the mature seeds.

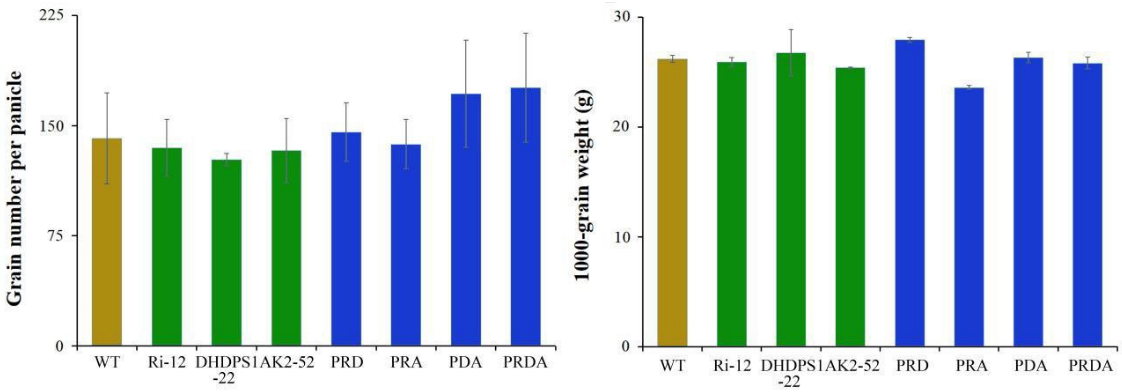

Figure S2. Analysis of grain number per ear and the 1000-grain weight of transgenic and wild-type (WT) rice grown in Yangzhou in 2018.

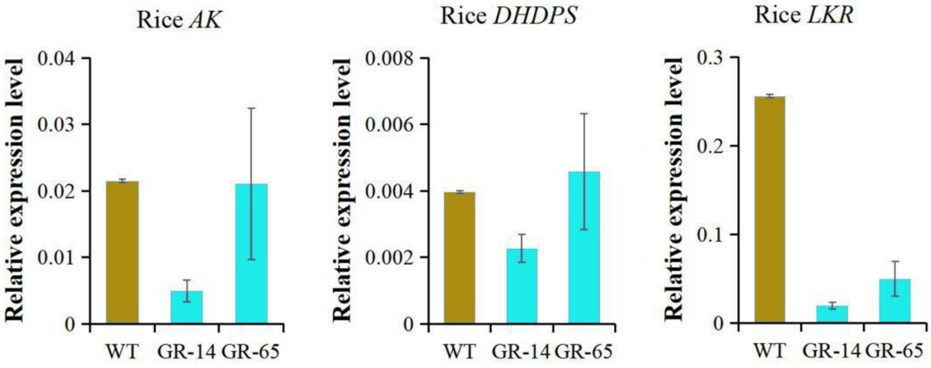

Figure S3. Effect of bacterial *AK* and *DHDPS* expression on expression of *AK* and *DHDPS* genes in rice.

Supplementary Tables

Table S1. RVA properties of rice flour and starch from the transgenic and WT rice.

Sample	Line	Peak 1	Trough 1	Breakdown	Final Visc	Setback	Peak Time	Pasting Temp
Rice milled flour	WT	3430	2325	1105	3679	249	6.1333	75
	Ri-12	2850	1828	1022	3151	301	6	74.95
	DHPDS1-22	3109	2197	912	3567	458	6.4	75.7
	AK2-52	3071	2302	769	3405	334	6.6	74.85
	PRD	3210	2061	1149	3466	256	6.0667	75.7
	PRA	3234	2319	915	3390	156	6.4667	74.9
	PDA	3492	2464	1028	3616	124	6.5333	74.15
	PRDA	3477	2124	1353	3142	-335	6.2	73.2
Starch	WT	1509	689	820	887	198	5.9333	76.45
	PRD	1565	873	692	1135	262	6.0667	74.95
	PRA	1504	508	996	651	143	5.6	74.2

Table S2. Construction of chimeric gene and pyramiding lines.

Transgenic lines	Constructs	Note
Ri-12	Gt1::LKR-RNAi	
DHDPS1-22	Gt1::DHDPS1	
AK2-52	Gt1::AK2	Rice endogenous modified AK2/DHDPS1 gene, Polygenic pyramid by hybrid
PDA	Gt1::DHDPS1×Gt1::AK2	
PRA	Gt1::LKR-RNAi×Gt1::AK2	
PRD	Gt1::LKR-RNAi×Gt1::DHDPS1	
PRDA	Gt1::LKR-RNAi×Gt1::AK2×Gt1::DHPS1	
GR-65	Gt1::Bacterial AK+Gt1::Bacterial	Bacterial AK and DHPS
GR-14	DHDPS+Gt1::LKR-RNAi	

Table S3. List of primers used in this study.

Primer name	Nucleotide sequence (5'→3')
LKR-R	CCTTAGCTGAGGCCAATCTAG
NOS-2R	ATTGCGGGACTCTAATCATAAAAAC
DHPS11-R	CGTAGTAAGGATTGATGTGG
AK-R	CGAACTTCATCACCACGCTCA
GT1	GCATTCAGTTCATTAGTCCT
Akrt-F	CTGACACTTCGGACCACTGACTACC
Akrt-R	ACACCATCAACATCTTTCCATACCTG
DHPSrt-R	CCCTGCTCAGTTGCGTGAATAG
DHPSrt-F	GTAGTGAAGTGAAAAATCGGACATC
LKRrt-F	ACTCTTCAATGCTTGTAACATCTCC
LKRrt-R	AGTAGGGTTGCTTGGTGCTTT
